# Clinicopathologic significance of legumain overexpression in cancer: a systematic review and meta-analysis

**DOI:** 10.1038/srep16599

**Published:** 2015-11-26

**Authors:** Ye Zhen, Guo Chunlei, Shen Wenzhi, Zhao Shuangtao, Luo Na, Wang Rongrong, Luo Xiaohe, Niu Haiying, Luo Dehong, Jiang Shan, Tan Xiaoyue, Xiang Rong

**Affiliations:** 1 Department of Tumor Molecular Biology, Nankai University School of Medicine, Tianjin 371000, China

## Abstract

Since reports on the clinical significance of legumain in cancer have shown inconsistent results, we systematically evaluated clinical indicators of legumain in cancer. We searched the Cochrane Library, PubMed, Embase, and EBSCO databases and the Wangfang and CNKI databases in China by using “legumain” and (“neoplasms” OR “cancer”) as search terms. We included case-controlled studies of legumain and cancer. The quality of the studies was evaluated by using Lichtenstein’s guidelines, and valid data was extracted for analysis. In total, 10 articles were included in this study. Meta-analysis showed that legumain was overexpressed in cancer compared with in normal tissue and was higher in stage III–IV disease than in I–II disease. Moreover, legumain overexpression was correlated with poor prognosis and clinical stage. Furthermore, Cancer Genome Atlas data showed that among patients with rectal cancer, those with tumors overexpressing legumain had shorter overall survival than those in the low expression group (P < 0.05). Legumain appears to be involved in tumor development and deterioration; thus, it can potentially be developed into both a marker for monitoring and diagnosing tumors and a therapeutic target.

Legumain (*LGMN*) is a cysteine endopeptidase that belongs to peptidase family C13^1^ and specifically hydrolyzes substrate asparaginyl bonds^2^ .Legumain was originally found in plants^3^ ; in mammals, LGMN was first identified in pigs as a 34-kDa glycoprotein^2^. The gene is widely conserved and is expressed in plants, parasites, and mammals, including humans^4^. In mouse kidneys, LGMN is mainly distributed in the proximal tubule^5^. Legumain shows the highest expression in lysosomes, but is also expressed in the extracellular environment^6^. However, in colorectal cancer cell lines, 13–17% of total LGMN is found in the nucleus^7^. An increasing number of studies have examined legumain, particularly its expression in tumors.

Alginates can accelerate the autocatalytic activation of pro-LGMN at pH 4.0 and 5.0^8^, with their activities increasing upon exposure to doxorubicin and reducing following knock-down of *p53*^9^. Similarly, intracellular LGMN activity is decreased by internalization of cystatin E/M^10^. Aza-peptidyl inhibitors containing various non-natural amino acids and different electrophilic active sites can inactivate LGMN^11^. Legumain can promote cell proliferation independently of endopeptidase activity^12^. The *LGMN* promoter is sensitive to nuclear Ca^2+^, which has been shown to regulate LGMN expression through Elk-1, affecting cell proliferation^12^.

Little or no LGMN is expressed in normal tissues^13^, including normal tissues adjacent to tumor tissue^4^. However, in solid tumors, LGMN is overexpressed in tumors, the matrix, and endothelial cells in the tumor microenvironment^14^. Briggs *et al.* reported that cystatin E/M can inhibit LGMN activity and the invasive potential of human melanoma cells^15^. Yoshio *et al.* reported that LGMN decreases the invasiveness and aggressiveness of prostate cancer^16^. Additionally, an LGMN DNA vaccine showed a potential protective effect against breast cancer in mice^17^. The results of studies on the clinical significance of LGMN expression in cancer are contradictory. To clarify the clinical and pathological significance of LGMN in cancer, we conducted a comprehensive meta-analysis of all eligible case-controlled studies. We also used The Cancer Genome Atlas (TCGA) (http://cancergenome.nih.gov/) database to confirm our results.

## Results

### Characteristics of eligible studies

We searched the Cochrane Library, PubMed, Embase, and EBSCO databases and the Wangfang and CNKI databases in China. After reviewing titles and abstracts and analyzing the articles in detail, a total of 10 relevant articles were included in this meta-analysis,^6^,^13^,^18^,^19^,^20^,^21^,^22^,^23^,^24^,^25^ seven of which were in English^13^,^18^,^19^,^21^,^22^,^23^ and three of which were in Chinese (Table 1)^20^,^24^,^25^. One of the articles compared LGMN expression in ovarian cancer with that in both normal tissues and benign tumors^23^. Although the definitions of LGMN overexpression (LGMN^High^) varied among the articles, most characterized LGMN expression in ≥25% of neoplastic cells as LGMN overexpression.

### Meta-analyses

#### Cancer tissues vs normal tissues

**Figure 1:**
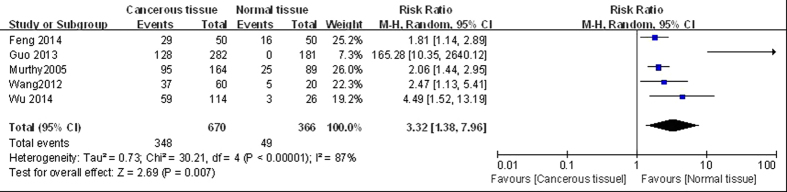
Forest plot of risk ratios of legumain overexpression in cancer tissue vs normal tissue groups.

Five articles compared LGMN^High^ in cancer tissue and in normal tissue^13^,^19^,^20^,^22^,^23^, including a total of 670 cancer cases (LGMN^High^: 51.9%) and 366 normal tissue samples (LGMN^High^: 13.4%). Since the studies were significantly heterogeneous (*P* < 0.10, *I*^2^ = 87%), we used a random-effect model for pooled analysis, which revealed significant differences between tumor tissues and normal tissues (*RR* = 3.32, 95% CI: 1.38–7.96, *P* < 0.05; Fig. 1). We analyzed each of the five articles individually. All five studies reported that LGMN^High^ rates in cancer tissue were higher than in normal tissue. However, the sample sources were different and the research methods were not completely consistent, likely leading to heterogeneity (*P* < 0.10, *I*^2^ = 87%). Overall, in our meta-analysis, the LGMN^High^ rate in cancer tissue was higher than that in normal tissue.

#### Good differentiation vs moderate and poor differentiation

**Figure 2:**
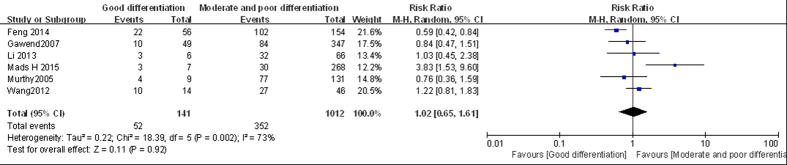
Forest plot of risk ratios of legumain overexpression in well-differentiated vs moderate–poorly differentiated tumor groups.

Six studies compared LGMN overexpression in well-differentiated (36.9%; 141 cases) and moderate–poorly differentiated (34.8%; 1012 cases) cancers^6^,^18^,^20^,^21^,^22^,^23^. Since the studies were significantly heterogeneous (*P* < 0.10, *I*^2^ = 73%), we used the random-effect model for pooled analysis. The results showed no significant difference between the two groups (*RR* = 1.02, 95% CI: 0.65–1.61; *P* > 0.05; Fig. 2). All six articles were included in this research; only two reported a significant difference in LGMN^High^ rates between the well-differentiated group and moderate–poorly differentiated groups. One study reported that LGMN^High^ rates in the well-differentiated group was lower than in the moderate–poorly differentiated group^20^. However, the other study showed different results^6^. The remaining four articles reported no significant difference in LGMN^High^ rates between the two groups. Therefore, combined with our meta-analysis, the LGMN^High^ rate in the well-differentiated group was similar to that in the moderate–poorly differentiated group.

#### N0 vs N+

**Figure 3:**
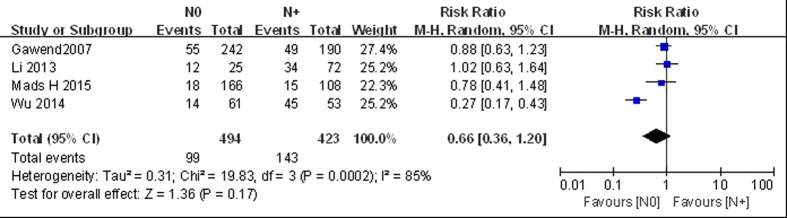
Forest plot of risk ratios of legumain overexpression in N0 vs N+ groups.

Four articles compared LGMN^High^ rates in patients without (N0; 20.0%; 494 cases) and with (N+; 33.8%; 423 cases) lymph node metastases^6^,^13^,^18^,^21^. Since the studies were significantly heterogeneous (*P* < 0.10, *I*^2^ = 85%), we used the random-effect model for pooled analysis. The results revealed no significant differences between the two groups (*RR* = 0.66, 95% CI: 0.36–1.20; *P* > 0.05; Fig. 3). Thus, this analysis showed that the LGMN^High^ percentage in the N0 group was similar to that in the N+ group.

#### Necrosis <10% vs necrosis >10%

Two articles compared the LGMN^High^ rates in cancers with <10% necrotic tissue (34.5%; 168 cases) and with >10% necrotic tissue (80.7%; 192 cases)^20^,^22^. Since the studies were significantly heterogeneous (*P* < 0.10, *I*^2^ = 97%), we used the random-effect model for pooled analysis, but found no significant difference between the two groups (*RR* = 0.40, 95% CI: 0.09–1.76; *P* > 0.05). One study found a significant difference in LGMN^High^ rates between the two groups^20^, but the other study did not^22^. According to the results of our meta-analysis, the LGMN^High^ percentage in the cancer with <10% necrotic tissue group is likely no higher than that in the >10% necrotic tissue group.

#### Male vs female

Five articles compared LGMN^High^ percentages in cancers in male patients (40.0%; 490 cases) and female patients (32.6%; 313 cases)^6^,^13^,^20^,^21^,^25^. Since the studies were not significantly heterogeneous (*P* > 0.10, *I*^2^ = 34%), we used the fixed-effect model for pooled analysis and found no significant difference between the two groups (RR = 1.10; 95% CI: 0.91–1.31; *P* > 0.05). This meta-analysis showed that among patients with cancer, the LGMN^High^ rate in men was not higher than in women.

#### Stage I−II vs stage III −IV disease

**Figure 4:**
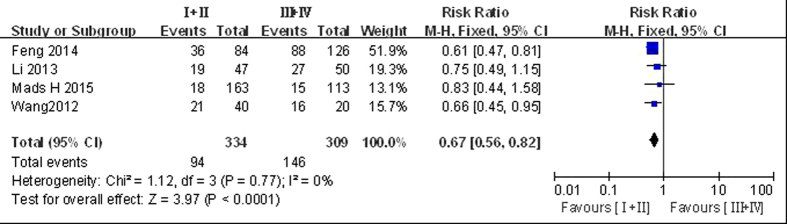
Forest plot of risk ratios of legumain overexpression in I–II vs III–IV groups.

Four articles compared LGMN^High^ rates in stage I−II disease (28.1%, 334 cases) and stage III −IV disease (47.2%; 309 cases)^6^,^20^,^21^,^23^. Since the studies were not significantly heterogeneous (*P* = 0.77, *I*^2^ = 0%), we used the fixed-effect model for pooled analysis and found a significant difference between the two groups (RR = 0.67, 95% CI: 0.56–0.82, *P* < 0.05; Fig. 4), with a higher LGMN^High^ rate in the stage III −IV group than in the stage I−II group.

#### Survival rate

**Figure 5:**
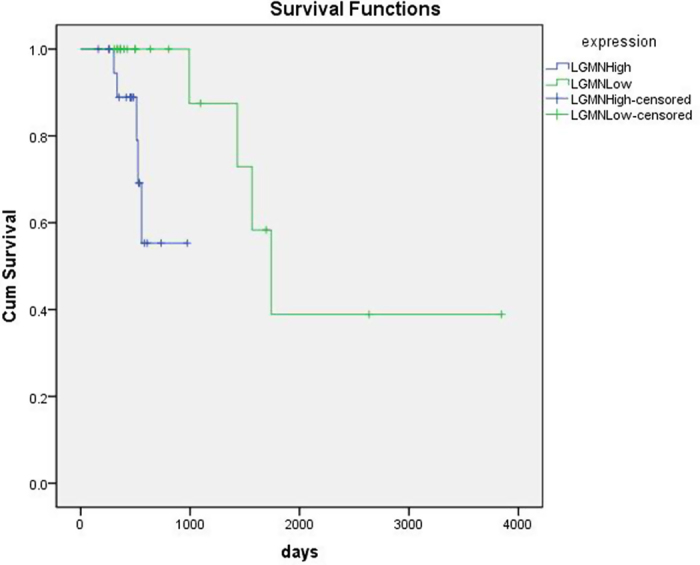
Kaplan–Meier survival curve of TCGA data comparing high legumain expression group with low legumain expression group.

Three articles compared the 5-year survival rates between patients with LGMN^High^ expression (51.7%; 298 cases) and those with LGMN^Low^ expression (79.9%; 154 cases)^13^,^19^,^24^. Since the studies were not significantly heterogeneous (*P* > 0.10, *I*^2^ = 0.0%), we used the fixed-effect model for pooled analysis and found a significant difference between the two groups (*RR* = 0.66; 95% CI: 0.57–0.76; *P* < 0.05). We collected 61 cases of patients with rectal cancer from the TCGA database. We analyzed the follow-up data and tumor mRNA data (HiSeq RNASeqV2). Kaplan–Meier survival analysis revealed that the LGMN^High^ group had significantly shorter survival compared to the LGMN^Low^ group (*P* < 0.05; Fig. 5).

#### Sensitivity analysis

Even after eliminating one study for a low-quality grade, LGMN expression was significantly different between cancer tissues and normal tissues. Compared with the random-effect model, fixed-effect model analysis showed *P* < 0.05 in the N0 vs N+ groups and necrosis <10% vs necrosis >10% groups, indicating unstable results. However, we found no significant differences in the *P* values in other groups, indicating that the results were stable.

#### Test of publication bias

**Figure 6:**
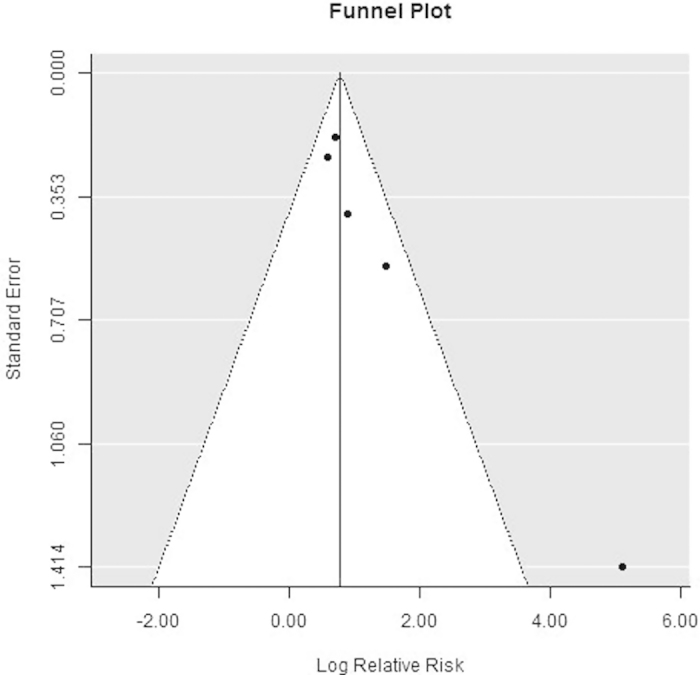
Funnel plot of risk ratios of legumain overexpression in cancer tissue vs normal tissue groups.

**Figure 7:**
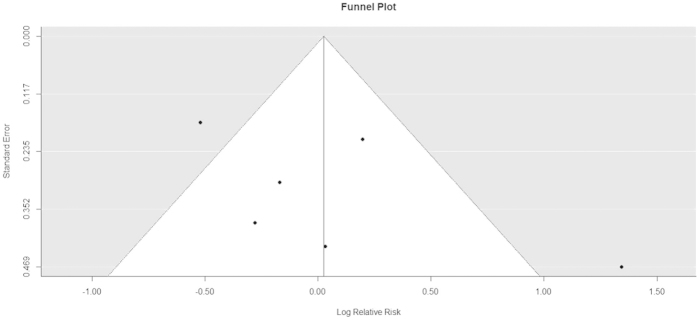
Funnel plot of risk ratios of legumain overexpression in well-differentiated vs moderate–poorly differentiated tumor groups.

In funnel plots of cancer tissue vs normal tissue (Fig. 6), Egger’s test showed *P* < 0.05 and Begg’s test showed *P* > 0.05. We considered Begg’s test to be more appropriate because the data was heterogeneous. The plots for well vs moderate–poor differentiation (Fig. 7) and male vs female showed an even and symmetrical distribution (Egger’s and Begg’s tests: *P* > 0.05 for both), indicating no publication bias.

## Discussion

This is the first meta-analysis to examine the association between LGMN and clinicopathological factors of cancer. We included ten studies in this analysis; three studies were in Chinese^20^,^24^,^25^ and two were doctoral theses^24^,^25^. One study included patient specimens from a biotechnology company^20^. Specimens used in the other nine studies were from surgical patients. Patient specimens had been pathology confirmed. We found differences in the sample sources and the research methods were not completely consistent. This may have led to heterogeneity (*P* ≤ 0.1, *I*^2^ ≥ 50%). The scores for two papers were low^6^,^20^, mainly because of poor case selection and bias.

Our analysis showed that the LGMN^High^ percentages in cancer tissues and normal tissues were significantly different, with values of 51.9% and 13.4% respectively, indicating that LGMN was overexpressed in tumors. Sensitivity analysis confirmed that the results were stable and reliable. The rank correlation test for funnel plot asymmetry showed *P* > 0.05, indicating no publication bias (Fig. 6). Legumain has been found to be overexpressed in tumor and tumor-associated cells^10^, mouse kidney tumors^26^, gastric cancer^19^,^20^, ovarian cancer^23^, and colorectal cancer^6^,^22^. Legumain was also found to be overexpressed in adult zebrafish at lesion sites after injury^27^, suggesting that it was a stress protein. Overexpression of LGMN has also been suggested to affect liver carcinogenesis^12^. Additional studies are required to determine how legumain promotes tumor cell proliferation.

Our meta-analysis demonstrated that LGMN overexpression was not related to tumor differentiation or to lymph node metastasis. Six studies compared LGMN overexpression in well-differentiated and moderate–poorly differentiated cancers. One study^20^ reported LGMN^High^ rates in well-differentiated tumors were lower than those in moderate–poorly differentiated tumors. However, one study showed different results^6^. The remaining four studies reported that LGMN overexpression was not related to tumor differentiation. Rong’s^25^ doctoral thesis also demonstrated that the overexpression of LGMN was not associated with tumor differentiation. Additionally, sensitivity analysis showed that this result was stable. Our analysis also showed that LGMN was not associated with tumor differentiation. Four studies compared LGMN^High^ percentages in patients without (N0) and with (N+) lymph node metastases. The results showed no significant difference between the two groups (*P* > 0.05). However, sensitivity analysis showed significant differences in *P* values, indicating that this result was not stable.

We found that the LGMN^High^ percentages were not significantly different in tumors with <10% necrotic tissue compared to in tumors with >10% necrotic tissue. The LGMN^High^ percentages also did not differ based on patient sex, suggesting that LGMN overexpression was not affected by hormones.

Our study also indicated that LGMN^High^ percentages were associated with clinical stage, further supporting a possible function for LGMN as a stress protein. Sensitivity analysis confirmed that this conclusion was stable. Other studies have demonstrated an association between LGMN and tumor invasion and metastasis. Li and colleagues reported that LGMN overexpression in tumor tissue was associated with large tumor size (*P* < 0.01)^21^, while Guo *et al.* showed that LGMN overexpression was associated with hepatic metastasis (*P* = 0.014)^19^. Cells overexpressing LGMN were found to be more migratory and invasive *in vitro*^4^, indicating that LGMN expression affects metastasis and invasion. Legumain can promote cell proliferation^12^, which may be related to its effect on tumor proliferation. Knock-down of *LGMN* decreased cell proliferation, but did not appear to promote cell apoptosis. Knock-down of *LGMN* in a mouse tumor model significantly reduced tumor growth and metastasis^28^, further supporting LGMN as a potential therapeutic target. Legumain can activate zymogene progelatinase A, which mediates extracellular matrix degradation^29^, this interaction may affect tumor invasion and metastasis^4^ .TBX2 is an oncogenic transcription factor that can drive breast cancer proliferation by maintaining LGMN activity^30^. The mechanism by which LGMN affects cell proliferation requires further study. Patients overexpressing LGMN showed lower 5-year survival rates than did patients with low LGMN expression, which agrees with results of reports correlating LGMN overexpression with poor prognosis in cancer^6^,^13^,^19^,^20^,^22^,^23^,^24^. TCGA data showed that among patients with rectal cancer, the LGMN^High^ group had significantly shorter survival than the LGMN^Low^ group (P < 0.05), further supporting the associations between high legumain expression, tumor progression, and poor prognosis.

Our results showed that LGMN was overexpressed in tumor tissues, affected cancer development and deterioration, and was associated with poor prognosis in cancer patients. We speculated that LGMN was a stress protein. Our meta-analysis showed that LGMN overexpression percentages were no higher in the N+ group than in the N0 group; however, sensitivity analysis indicated that this conclusion was unstable. Considering that LGMN was involved in tumor development and deterioration, we doubted that LGMN was also associated with lymph node metastasis, as it was an indicator of increased malignancy. We also hypothesized that LGMN was not related to tumor differentiation, tumor tissue necrosis, or patient sex. Our analysis data support these hypotheses. These factors share a common characteristic in that they do not effect tumor progression, suggesting that LGMN’s function in cancer was limited to tumor progression, unless other mechanisms were involved. Additionally, LGMN was not related to patient age, tumor location, or body mass index, as these factors were found to be unrelated to tumor progression.

Legumain is widely conserved, expressed in both plants and animals, and has unique distribution characteristics^4^,^6^,^7^,^16^. Although low expression of LGMN is observed in normal tissue^13^, it is overexpressed in tumor, matrix, and endothelial cells in the tumor microenvironment^14^. Legumain overexpression in rectal cancer offers both an opportunity and a challenge as a therapeutic target^6^. Knock-down of *LGMN* was shown to control tumors^28^, further supporting that LGMN can be used as a therapeutic target for cancer diagnosis and therapy^31^. Chen *et al.* developed an MRI contrast agent and a near-infrared fluorescence probe to monitor LGMN activity in tumors^32^. Numerous studies have been conducted to develop an LGMN vaccine for tumor treatment^17^,^33^,^34^ . As the target of a multi-peptide vaccine in a breast cancer model, legumain effectively inhibited the tumor load^35^. We previously developed a drug carrier for tumor treatment based on the attachment of endoprotease to LGMN^36^. The TBX2–CST6–LGMN signaling pathway may be a target for the development of novel therapies against breast cancer^30^. U.S. President Barack Obama unveiled a new medical research initiative known as the Precision Medicine Initiative on Jan 30, 2015^37^. Targeting of LGMN should be involved in this initiative.

## Conclusion

Our findings indicated that LGMN was overexpressed in cancer and was associated with tumor progression, deterioration, and poor prognosis in cancer. Overexpression of LGMN was not associated with tumor differentiation, tumor tissue necrosis, or patient sex. LGMN may be used as a diagnostic marker or therapeutic target.

## Methods

This meta-analysis was conducted in accordance with the Preferred Reporting Items for Systematic Reviews and Meta-Analyses (PRISMA) guidelines^38^.

### Study identification and eligibility criteria

We systematically searched the Cochrane Library, PubMed, Embase, and EBSCO databases and the Wangfang and CNKI databases in China by using the following search terms: “legumain” and (“neoplasms” [MeSH Terms] or “cancer”). We included studies published up to July 2015 with no language limitations. A study was eligible in the meta-analysis if it: (1) investigated the association between LGMN and cancer; (2) cancer cells were acquired from humans; (3) LGMN was tested by using immunohistochemical methods; (4) provided sufficient data on the relationship between LGMN expression and clinicopathological variables. We excluded studies that (1) had no control groups; (2) used animal models; (3) were case reports, letters, comments, or review articles; (4) did not allow us to extract needed data; (5) did not conduct immunohistochemical method detection of LGMN; (6) included patients who had received chemotherapy or radiotherapy before the study; or (7) included duplicated data. We also downloaded follow-up data and tumor mRNA data (HiSeq RNASeqV2) for patients with rectal cancer from the TCGA database on April 10th, 2015. After eliminating records with incomplete data, we sorted the remaining 61 cases into three groups based on LGMN expression: 21 studies with the highest expression (LGMN^High^), 20 with the lowest expression (LGMN^Low^), and 20 with moderate expression (moderate expression group) (Supplemental file 1).

### Data extraction

For each study, two authors independently extracted the first author, publication year, country, numbers of cases and controls, cancer types, LGMN expression, and clinical indicators. Disagreements were resolved by discussion between the two investigators.

### Quality score assessment

The quality of the studies was evaluated by the Lichtenstein’s guidelines for case-control studies^39^. We evaluated quality based on the following aspects to determine whether: (1) the study design was used the scientific method; (2) inclusion criteria and basic structure characteristics of the studies were clear; (3) processing factors and methods were correct; (4) statistical methods were correct; (5) the existence of bias in the research was discussed. Satisfaction of any one of the above 5 aspects earned 1 point. When a study earned three points or more, the quality was considered reliable. Study quality was evaluated by two independent researchers (Zhao Shuangtao and Ye Zhen). Disagreements between the two researchers were resolved through discussion.

### Statistical analysis

We used RevMan 5.2 software, provided by the Cochrane Collaboration, to perform the meta-analysis and to merge *RR* values. Heterogeneity was tested by chi-square analysis. If *P* ≤ 0.10 for the chi-square test indicated heterogeneity across studies, a fixed-effects model was used for homogeneous data (*P* > 0.1, *I*^2^ < 50%) and a random effects model for significantly different data (*P* ≤ 0.1, *I*^2^ ≥ 50%). Publication bias was evaluated by using a funnel plot if a sufficient number of studies (*n* > 4) was included. We used the Egger’s and Begg’s tests to evaluate publication bias by using R version 3.1.2 (http://www.r-project.org/). Stability of the data was tested by sensitivity analysis. We eliminated a low-quality document after sensitivity analysis. We also compared the use of the fixed-effect model and random-effect model for sensitivity analysis. We created a Kaplan–Meier survival curve by using the log-rank test for TCGA data to compare the LGMN^High^ group with the LGMN^Low^ group. SPSS 22.0 (SPSS, Inc., Chicago, IL, USA) was used for this statistical analysis. P < 0.05 was considered significant.

## Additional Information

**How to cite this article**: Zhen, Y. *et al.* Clinicopathologic significance of legumain overexpression in cancer: a systematic review and meta-analysis. *Sci. Rep.* **5**, 16599; doi: 10.1038/srep16599 (2015).

## Supplementary Material

Supplementary Information

## Figures and Tables

**Table 1:** Characteristics of included studies.

Study	Region	Tumor site	Main methods	Observe	Score
Murthy 2005	Sweden	Colorectal	Immunohistochemistry Western blotting	Primary Tumor vs Adjacent Normal Mucosa Differentiation: Good vs Moderate vs Poor Necrosis: <10% vs >10%	4
Gawend 2007	Germany	Breast	Immunohistochemistry	StagepN: Negative vs Positive Grade: 1 vs2 vs3	4
Li 2010	China	Eyes	Immunohistochemistry	Survival rates	4
Rong 2010	China	Eyes	Immunohistochemistry	Female vs male	4
Wang 2012	China	Ovarian	Immunohistochemical assay Quantitative RT-PCR Western blotting	Malignant tissues vs Normal tissues Histological grad:1 vs2 vs3 Stage :I+II vs III+IV	4
Guo 2013	China	Gastric	Immunohistochemistry Western blotting Real-time PCR	Primary Cancer Tissue vs Adjacent Normal Mucosa Female vs male T Stage :T1 +T2 vs T3 vsT4 N Stage:N0 vsN1 +N2+ N3	4
Li 2013	China	Gastric	RNA interference Western blotting Quantitative real-time PCR Immunohistochemistry	Female vs Male TNM stages:I+ II vs III +IV Regional lymph nodes:N0 vs N1+ N2+N3	4
Feng 2014	China	Gastric	Immunohistochemistry	Primary Tumor vs Normal Mucosa Female vs Male TNM stages:I+ II vs III +IV Differentiation: Good vs Moderate vs Poor Necrosis: <10% vs >10%	3
Wu 2014	China	Breast	Immunohistochemistry	Primary Tumor vs Normal Mucosa Female vs Male N Stage:N0 vsN1 +N2+ N3	4
Haugen 2015	Norway	Colorectal	Immunohistochemistry	Female vs Male Regional lymph nodes:N0 vs N1+ N2+N3 Differentiation: Good vs Moderate vs Poor TNM stages:I+ II vs III +IV	3

N0: lymph node metastasis negative group. N1,2,3,4: lymph node metastasis positive group.
