# Supplementary material for: Clinicopathologic significance of legumain overexpression in cancer: a systematic review and meta-analysis
Source: Sci Rep. 2015 Nov 26;5:16599. doi: 10.1038/srep16599 (PMC4660395; doi:10.1038/srep16599)
Supplement: Supplementary Information [file srep16599-s1.docx]

Supplementary materials for

“Clinicopathologic significance of legumain overexpression in cancer: a systematic review and meta-analysis”

Ye Zhen, Guo Chunlei, ShenWenzhi, Zhao Shuangtao,Luo Na, Wang Rongrong, Luo Xiaohe, Niu Haiying, Luo Dehong, Jiang Shan, Tan Xiaoyue*, Xiang Rong*

Department of Tumor Molecular Biology, Nankai University School of Medicine, Tianjin 371000, China

***Corresponding author:** Xiang Rong, Department of Tumor Molecular Biology, Medical School of Nankai University, 94 Weijin Road, Tianjin 300071, China. Tel.: +86 22 23504447; fax: +86 22 23502554; email: [rxiang@nankai.edu.cn](mailto:rxiang@nankai.edu.cn)

Dr. Tan Xiaoyue, Department of Immunology, The School of Medicine, Nankai University, Tianjin, China; 300071, Tianjin, China, [xiaoyuetan@nankai.edu.cn](mailto:%20xiaoyuetan@nankai.edu.cn)

Table S1 The survival follow-up data and tumor mRNA data (HiSeq RNASeqV2) of legumain for patients with rectal cancer from the TCGA database.

| Id | Survival time(days) | Vital status | Normalized counts |
| --- | --- | --- | --- |
| TCGA-DY-A1DE | 3932 | Alive | 2028.68 |
| TCGA-DY-A0XA | 3846 | Alive | 1688.0624 |
| TCGA-DY-A1H8 | 992 | Dead | 1293.5954 |
| TCGA-EI-7004 | 257 | Alive | 2929.8701 |
| TCGA-EI-7002 | 364 | Alive | 1508.355 |
| TCGA-EI-6917 | 531 | Alive | 3833.2709 |
| TCGA-EI-6885 | 415 | Alive | 2665.9368 |
| TCGA-EI-6884 | 328 | Alive | 2258.1655 |
| TCGA-EI-6883 | 350 | Alive | 5074.3108 |
| TCGA-DC-6154 | 365 | Alive | 1523.3635 |
| TCGA-DC-6156 | 457 | Alive | 4236.4461 |
| TCGA-AG-3732 | 1003 | Alive | 1933.3333 |
| TCGA-DC-6158 | 334 | Dead | 3586.88 |
| TCGA-DC-6157 | 1095 | Alive | 1241.8791 |
| TCGA-DC-5869 | 395 | Alive | 1879.4018 |
| TCGA-DC-6160 | 914 | Alive | 2068.7294 |
| TCGA-DC-5337 | 457 | Alive | 3017.2486 |
| TCGA-AF-5654 | 512 | Dead | 4676.8226 |
| TCGA-AF-2687 | 734 | Alive | 5213.708 |
| TCGA-AF-2690 | 524 | Dead | 5098.2298 |
| TCGA-AF-2693 | 581 | Alive | 3202.8562 |
| TCGA-AF-3911 | 1148 | Alive | 1799.5074 |
| TCGA-AF-4110 | 441 | Alive | 2175.4605 |
| TCGA-AF-6136 | 468 | Alive | 2619.3467 |
| TCGA-DC-6682 | 397 | Alive | 1546.1032 |
| TCGA-DC-6683 | 306 | Alive | 1448.1782 |
| TCGA-DT-5265 | 161 | Alive | 3180.9476 |
| TCGA-F5-6571 | 952 | Alive | 2104.2997 |
| TCGA-F5-6702 | 452 | Alive | 3167.2862 |
| TCGA-G5-6235 | 1696 | Alive | 1493.1148 |
| TCGA-DY-A1DC | 1258 | Dead | 1967.89 |
| TCGA-DY-A1DD | 1741 | Dead | 712.6982 |
| TCGA-AF-6655 | 609 | Alive | 2516.695 |
| TCGA-AF-6672 | 316 | Alive | 1903.3478 |
| TCGA-BM-6198 | 310 | Alive | 2245.2302 |
| TCGA-CL-4957 | 377 | Alive | 1774.7527 |
| TCGA-DC-4749 | 335 | Alive | 1316.9761 |
| TCGA-DC-6155 | 425 | Alive | 1695.2184 |
| TCGA-EI-6506 | 625 | Alive | 2138.0569 |
| TCGA-EI-6507 | 607 | Alive | 2351.1013 |
| TCGA-EI-6508 | 636 | Alive | 1389.4749 |
| TCGA-EI-6509 | 517 | Alive | 1907.8404 |
| TCGA-EI-6510 | 556 | Alive | 2353.2423 |
| TCGA-EI-6511 | 482 | Alive | 3628.0147 |
| TCGA-EI-6882 | 262 | Alive | 2512.8645 |
| TCGA-EI-6512 | 538 | Alive | 2827.1819 |
| TCGA-EI-6513 | 497 | Alive | 1947.4504 |
| TCGA-EI-6514 | 496 | Alive | 1365.6374 |
| TCGA-F5-6464 | 303 | Dead | 4129.1114 |
| TCGA-F5-6465 | 715 | Alive | 2492.5916 |
| TCGA-G5-6233 | 556 | Dead | 2869.1099 |
| TCGA-G5-6572 | 1432 | Dead | 1462.4394 |
| TCGA-DY-A1DG | 1566 | Dead | 1275.1349 |
| TCGA-AG-3902 | 974 | Alive | 3884.2592 |
| TCGA-DC-6681 | 394 | Alive | 2013.073 |
| TCGA-DC-4745 | 334 | Alive | 893.7044 |
| TCGA-G5-6641 | 804 | Alive | 1606.5235 |
| TCGA-EI-6881 | 499 | Alive | 1252.052 |
| TCGA-AF-A56L | 2007 | Alive | 2131.6981 |
| TCGA-AF-A56N | 360 | Alive | 1240.5985 |
| TCGA-AF-A56K | 2635 | Alive | 1571.6862 |

|  |
| --- |
